# In-hospital outcomes and documented 90-day readmission to the index hospital in older patients with Parkinson’s disease hospitalised for lower respiratory tract infection: a single-centre retrospective cohort study

**DOI:** 10.3389/fneur.2026.1861214

**Published:** 2026-09-16

**Authors:** Yue Zou, JinRong Li, SiWei Luo

**Affiliations:** 1 The Affiliated Hospital of Southwest Medical University, Luzhou, China; 2 The Fourth Affiliated Hospital of Southwest Medical University, Meishan, China

**Keywords:** hospital outcomes, hospital readmission, lower respiratory tract infection, older adults, Parkinson’s disease

## Abstract

**Background:**

Lower respiratory tract infection (LRTI) is a major cause of hospitalisation in older adults with Parkinson’s disease (PD), but it remains uncertain whether PD-related measures provide clinically relevant information beyond general vulnerability and acute infection severity.

**Methods:**

We conducted a single-centre retrospective cohort study of patients aged ≥65 years with neurologist-diagnosed PD who were hospitalised for LRTI between 2018 and 2025. The primary outcome was a composite of intensive care unit admission, invasive mechanical ventilation, or in-hospital death. Documented readmission to the index hospital within 90 days among survivors was analysed as a secondary exploratory outcome. Multivariable logistic and Cox regression models evaluated clinical factors associated with these outcomes. A hierarchical extension added Hoehn–Yahr stage and pre-admission wheelchair- or bed-bound status to the base clinical model. Calendar-period analyses and bootstrap internal validation were performed.

**Results:**

Among 246 patients, 62 (25.2%) experienced the composite in-hospital outcome. In the PD-specific extended model, higher Charlson Comorbidity Index (adjusted odds ratio [OR] per 1-point increase 1.19, 95% confidence interval [CI] 1.00–1.42), hypoxaemia (OR 2.00, 95% CI 1.01–3.96), serum albumin <35 g/L (OR 3.18, 95% CI 1.65–6.14), sepsis or septic shock (OR 2.41, 95% CI 1.17–4.98), and pre-admission wheelchair- or bed-bound status (OR 2.08, 95% CI 1.08–4.01) were associated with the outcome. Hoehn–Yahr stage, PD duration, tube feeding, and antiparkinsonian medication burden were not independently associated. Among 228 patients discharged alive, 74 (32.5%) had a documented readmission to the index hospital. Hypoalbuminaemia (adjusted hazard ratio [HR] 1.82, 95% CI 1.12–2.95) and non-home discharge (HR 1.96, 95% CI 1.19–3.24) were among the factors associated with documented same-hospital return. Bootstrap-corrected discrimination was 0.81 for the extended logistic model and 0.70 for the Cox model.

**Conclusion:**

Severe in-hospital deterioration was associated mainly with general physiological vulnerability, acute infection severity, and pre-admission mobility dependence, whereas most other available PD-specific measures showed no clear independent association. These findings support integrated clinical assessment but not a validated PD-specific prediction strategy; the post-discharge analysis reflects documented return to the index hospital rather than complete population-level readmission.

## 1 Introduction

Lower respiratory tract infection (LRTI) is a common cause of acute deterioration and hospitalisation in older adults with Parkinson’s disease (PD), but it remains uncertain whether Parkinson’s disease–related measures provide clinically relevant information beyond general vulnerability and the severity of the acute infection. Several features of PD may increase susceptibility to respiratory infection and complicate recovery, including impaired cough, postural abnormalities, autonomic dysfunction, and swallowing impairment (1), reduced mobility, and dependence on caregivers. These factors may promote aspiration, limit airway clearance, and reduce the ability to compensate for acute respiratory compromise (2). In older patients with advanced disease, LRTI may therefore lead not only to respiratory failure but also to delirium, acute kidney injury, prolonged immobility, hospital-acquired complications, functional decline, and death (3).

The clinical course of LRTI in PD is unlikely to be determined by neurological disease alone (4). Many patients are also affected by multimorbidity, frailty, cognitive impairment, previous hospitalisation, recurrent pneumonia, and reduced physiological reserve (5). During the acute infection, hypoxaemia, systemic inflammation, sepsis, renal dysfunction, and hypoalbuminaemia may further indicate severe illness or limited capacity to recover (6). The interaction between these general geriatric factors, PD-related disability, and the severity of the acute respiratory episode may influence the need for intensive care, invasive mechanical ventilation, and other forms of organ support (7). However, the relative contribution of these domains has not been clearly established in routine clinical practice.

Previous studies have often focused on pneumonia-related mortality, aspiration, dysphagia, or long-term survival in patients with PD (8). Although these outcomes are clinically important, less attention has been given to the broader in-hospital trajectory, including treatment intensity, acute complications, discharge disposition, and short-term return to hospital (9). In particular, it remains uncertain whether conventional measures of PD burden, such as Hoehn–Yahr stage, disease duration, tube feeding, or antiparkinsonian medication burden, provide meaningful information beyond general comorbidity, frailty, acute infection severity, and directly observed functional dependence (10, 11). Pre-admission wheelchair- or bed-bound status may be especially relevant because it reflects the combined consequences of motor impairment, reduced respiratory reserve, dependence in activities of daily living, and vulnerability to hospital-associated deconditioning.

Post-discharge outcomes are also important in this population. Older patients who survive a severe respiratory admission may leave the hospital with residual physiological instability, new functional limitations, increased care needs, or dependence on rehabilitation and long-term care services. Previous pneumonia-related hospitalisation, a complicated index admission, prolonged length of stay, and non-home discharge may therefore be associated with subsequent return to hospital (12). At the same time, readmission patterns may be influenced by local referral networks, transfer pathways, caregiver availability, and access to post-acute care. For this reason, documented return to the index hospital should be distinguished from complete population-level readmission (13).

Evidence addressing these questions in Chinese tertiary hospitals remains limited. Such institutions often manage older patients with advanced PD, multimorbidity, recurrent respiratory infection, and complex discharge needs, yet detailed data linking baseline vulnerability, acute illness severity, in-hospital management, and post-discharge outcomes are sparse. A structured evaluation of these factors may help clarify which routinely available clinical characteristics are most closely associated with short-term deterioration and whether PD-related measures remain associated after general vulnerability and acute illness severity are considered.

We therefore conducted a single-centre retrospective cohort study of older adults with neurologist-diagnosed PD who were hospitalised for LRTI at a tertiary teaching hospital in China. The primary objective was to evaluate factors associated with a composite adverse in-hospital outcome comprising intensive care unit admission, invasive mechanical ventilation, or in-hospital death. We first assessed general comorbidity, frailty, and acute infection severity and then examined whether PD-related characteristics, including Hoehn–Yahr stage, disease duration, pre-admission mobility status, tube feeding, and antiparkinsonian medication burden, remained associated with the primary outcome after these general and acute-illness factors were considered. Among patients discharged alive, we secondarily explored factors associated with documented readmission to the index hospital within 90 days.

## 2 Methods

### 2.1 Study design and setting

This was a single-centre, retrospective cohort study of older adults with Parkinson’s disease (PD) who were hospitalised with lower respiratory tract infection (LRTI) between 2018 and 2025. Because the study period encompassed major changes in healthcare delivery associated with the COVID-19 pandemic, calendar time was considered a potential contextual confounder. Admissions were categorised according to the date of the index hospitalisation as occurring during 2018–2019, 2020–2022, or 2023–2025. These categories were intended to capture broad temporal differences in admission and hospital-care practice and were not considered substitutes for individual-level information on SARS-CoV-2 infection or pandemic-related disruptions in care. Consecutive eligible admissions were identified through the hospital information system and followed from admission to discharge, and, among survivors, for 90 days after discharge. The primary in-hospital endpoint was a composite adverse outcome comprising ICU admission, invasive mechanical ventilation, or in-hospital death. Among patients discharged alive after the index LRTI admission, the secondary exploratory post-discharge endpoint was time to first documented readmission to the index hospital within 90 days. The study was conceived and reported as an observational cohort using a prespecified covariate and outcome framework, with no deviation from routine clinical pathways.

The cohort was assembled at a tertiary teaching hospital in China that provides emergency, general medical, respiratory, neurology, geriatric and ICU services for patients with PD. All diagnostic evaluations, antimicrobial choices, supportive treatments, and discharge dispositions were determined by the treating clinicians according to prevailing institutional practice and national guidelines; the research team had no role in clinical decision-making. The analysis relied exclusively on routinely collected clinical, laboratory, nursing and administrative data extracted from the electronic medical record and related hospital databases, and did not mandate any additional investigations or follow-up beyond usual care. The protocol was approved by the institutional ethics committee, which granted a waiver of individual informed consent because of the retrospective design and use of de-identified data.

### 2.2 Participants and eligibility criteria

Potentially eligible encounters were first identified by electronic search of the hospital information system for inpatient admissions in which Parkinson’s disease was listed as a pre-existing diagnosis and a lower respiratory tract infection was coded as the primary reason for hospitalisation during the study period. Patients were included if they were aged 65 years or older at admission, had a neurologist-documented diagnosis of idiopathic PD established before or at the index admission, and were admitted with LRTI as the principal diagnosis. Records indicating atypical Parkinsonism, drug-induced Parkinsonism, vascular Parkinsonism, or another secondary Parkinsonian syndrome were not considered to meet the PD eligibility criterion. When an individual experienced more than one LRTI-related admission within the study window, only the first admission fulfilling all criteria was retained and treated as the index hospitalisation, to avoid within-patient clustering and to ensure a reproducible unit of analysis.

We excluded admissions in which patients were younger than 65 years, the length of stay was less than 24 h, or the primary discharge diagnosis indicated that LRTI was not the main cause of the admission. Admissions in which the treatment goal was explicitly documented at or shortly after presentation as end-of-life or comfort-only care were excluded from the analytical cohort. In these patients, non-admission to the ICU and non-use of invasive mechanical ventilation reflected treatment limitation rather than absence of clinical deterioration, making the composite endpoint structurally non-comparable with that of patients eligible for treatment escalation. This exclusion was applied before outcome modelling. Formal do-not-resuscitate or do-not-intubate status outside these explicitly documented cases was not consistently retrievable from the retrospective records. Finally, 26 admissions were excluded because the available records remained insufficient, despite review of physician notes, nursing documentation, laboratory reports, imaging reports, and discharge summaries, to determine one or more variables required for eligibility or ascertainment of the primary in-hospital or secondary post-discharge endpoint. These exclusions reflected non-recoverable source-record incompleteness rather than isolated missing values in otherwise analysable records.

### 2.3 Data sources and variables

All data were abstracted retrospectively from the hospital’s electronic medical record, laboratory information system and administrative databases using a predefined electronic case report form. For each eligible index admission, clinicians trained in PD and respiratory medicine extracted information on baseline characteristics, comorbidities, clinical presentation, laboratory findings, in-hospital management, complications, discharge disposition and 90-day readmission status. When necessary, free-text physician notes and routine ward documentation, discharge summaries and radiology reports were reviewed to clarify diagnoses, functional status and the circumstances of the acute respiratory illness. All records were de-identified prior to analysis. A predefined source hierarchy was used for variables that could vary during acute illness. Pre-admission neurology assessments and documented baseline function were prioritised over observations made after acute clinical deterioration. When documentation was conflicting or insufficient to support a classification, the variable was not inferred solely from related diagnoses or treatments.

Baseline variables included age, sex, PD duration, and motor severity. Hoehn–Yahr stage was obtained preferentially from the most recent neurology assessment before the index admission. When only an admission-stage assessment was available, it was used only if the documentation explicitly indicated that the recorded stage represented the patient’s usual pre-illness state. Hoehn–Yahr stage was dichotomised as stages 1–2 versus stages 3–5. Parkinson’s disease–related functional dependence was represented by wheelchair- or bed-bound status during the usual pre-admission state; immobility developing only after the onset of the acute infection was not classified as baseline mobility dependence. Tube feeding at admission and treatment with three or more antiparkinsonian medications were considered additional markers of advanced disease burden and treatment complexity. Living situation (community dwelling vs. nursing home or long-term care facility), mobility and functional status (including wheelchair- or bed-bound status) and the presence of tube feeding or severe dysphagia were obtained from admission assessments and routine ward documentation. Comorbidities were ascertained from active problem lists, prior discharge diagnoses and specialist notes and summarised using the Charlson Comorbidity Index, with specific recording of chronic heart failure, chronic obstructive pulmonary disease, prior ischaemic or haemorrhagic stroke and dementia or cognitive impairment. Markers of recent clinical instability—at least one hospitalisation in the preceding 12 months, pneumonia-related hospitalisation within 12 months, at least one documented fall in the previous year and long-term home oxygen therapy—were also captured. At presentation with LRTI, vital signs (heart rate, respiratory rate and peripheral oxygen saturation on room air) were abstracted from the first set of observations in the emergency department or admission ward, together with laboratory parameters including white blood cell count, C-reactive protein, procalcitonin, serum creatinine, estimated glomerular filtration rate and serum albumin. Clinical frailty was graded retrospectively using the Clinical Frailty Scale on the basis of documented function during the period preceding the acute infection, rather than functional impairment developing during hospitalisation. Scores of 5 or higher denote at least moderate frailty. A score was not assigned when the available documentation was insufficient to distinguish baseline dependence from acute illness-related decline. Features of the index LRTI episode—classification as aspiration-related LRTI, presence of sepsis or septic shock at admission and initial use of supplemental oxygen—were obtained from admission and early progress notes. The calendar period was derived directly from the date of the index admission. Individual SARS-CoV-2 infection status and detailed information on pandemic-related visitor restrictions, caregiver availability, rehabilitation access, and institutional referral pathways were not consistently available throughout the study period. The clinical variables described above formed the basis for the descriptive comparisons and primary multivariable models, while the calendar period was incorporated into additional sensitivity analyses.

### 2.4 Definitions of infection phenotype, comorbidity, frailty and outcomes

Parkinson’s disease was defined as a neurologist-documented diagnosis of idiopathic PD established before or at the index admission. Documentation indicating atypical or secondary Parkinsonism was considered inconsistent with the PD eligibility criterion. The index hospitalisation was classified as being for lower respiratory tract infection when the primary discharge diagnosis was LRTI, pneumonia or bronchopneumonia, and this was supported by acute respiratory symptoms (e.g., cough, sputum, dyspnoea, or pleuritic chest pain), new or worsened auscultatory findings, compatible new infiltrates on chest radiography or computed tomography, and elevated inflammatory markers. Within this spectrum, an episode was classified as aspiration-related LRTI when the treating team explicitly documented aspiration pneumonia or when the record contained both: (1) a witnessed or strongly suspected aspiration event, established swallowing impairment, tube feeding, or advanced bulbar dysfunction; and (2) compatible new infiltrates in dependent lung segments. Advanced PD, dysphagia, or tube feeding alone was not considered sufficient to establish aspiration-related LRTI. This classification reflected routinely available clinical documentation rather than an independently adjudicated diagnostic reference standard. Sepsis or septic shock at admission was recorded only when explicitly documented by the admitting clinical team. Because the physiological and laboratory components required to retrospectively reconstruct Sepsis-3 criteria were not uniformly available throughout 2018–2025, sepsis status was not reclassified solely by the research team and was treated as a dichotomous routine-care diagnosis.

Comorbidity burden was summarised using the Charlson Comorbidity Index, calculated retrospectively from recorded diagnoses using standard weighting of component conditions. Clinical frailty was graded with the Clinical Frailty Scale (CFS) on the basis of pre-admission functional descriptions in physician documentation and routine ward records, and was dichotomised as CFS ≥ 5 versus <5 for analysis, with the higher group indicating at least moderate frailty. Baseline serum albumin was defined as the first value obtained within 24 h of admission; low albumin was operationalised as <35 g/L, consistent with conventional thresholds for hypoalbuminaemia in older inpatients. This variable was interpreted as hypoalbuminaemia and was not used as a diagnostic criterion for malnutrition. Other laboratory and physiological variables, including estimated glomerular filtration rate, were analysed in their continuous form.

The composite adverse in-hospital outcome was defined as the occurrence of at least one of the following during the index admission: ICU admission, invasive mechanical ventilation, or in-hospital death. The three components were not mutually exclusive and were also reported separately. Patients experiencing at least one component were classified as having the composite endpoint, whereas those experiencing none of the three components were classified as having no composite adverse in-hospital outcome. To examine whether the primary findings were dependent on institution-specific ICU admission practice, a harder sensitivity endpoint was defined as invasive mechanical ventilation or in-hospital death during the index admission. In-hospital complications—including delirium, acute kidney injury, hospital-acquired infection, pressure ulcers, in-hospital falls and objectively confirmed venous thromboembolism—were identified from clinician documentation, diagnostic codes and relevant investigation reports and were used as descriptive variables. Discharge disposition was categorised as home versus non-home, with the latter including transfer to a rehabilitation ward or hospital, a nursing or long-term care facility, or another acute-care hospital. For the secondary post-discharge analysis, documented 90-day readmission to the index hospital was defined as the first new inpatient admission to the study hospital for any cause within 90 days after discharge from the index LRTI hospitalisation. Patients without a documented return in the index hospital database were administratively censored at 90 days. Readmissions to other institutions and deaths occurring outside the index hospital were not captured by this endpoint.

### 2.5 In-hospital management and complications

In-hospital management variables were abstracted from electronic prescribing records, medication administration records, routine ward documentation, dietitian and rehabilitation notes, and intensive care flow sheets. Recorded measures included antimicrobial timing, initial regimen and treatment duration; respiratory and haemodynamic support; nutritional, swallowing, and rehabilitation interventions; in-hospital complications; length of stay; and discharge destination. Early mobilisation was defined as documented transfer out of bed to a chair or standing, with assistance when required, within 48 h of admission. Complications were based on diagnoses recorded by the treating teams and were not independently adjudicated. Discharge destination was categorised as home or non-home, including rehabilitation, long-term care, or transfer to another acute-care hospital.

### 2.6 Ascertainment of documented 90-day readmission to the index hospital

Post-discharge events were ascertained through the index hospital information system, which prospectively records inpatient encounters under unique patient identifiers. The discharge date of the index LRTI hospitalisation was defined as time zero. Subsequent inpatient admissions to the same institution within 90 days were identified by linkage within the hospital information system using name, national identification number, and medical record number before the linked dataset was de-identified for analysis. Admission records were reviewed to confirm that each event represented a new inpatient stay rather than an administrative transfer or an internal bed movement. The secondary exploratory endpoint was the first documented readmission to the index hospital for any cause within 90 days after discharge.

Patients without a documented readmission in the index hospital database were administratively censored at 90 days. No additional telephone, outpatient, regional health-database, or mortality-registry follow-up was available. Readmissions to other hospitals and post-discharge deaths occurring outside the index hospital could therefore not be systematically identified. Accordingly, the analysis estimated the hazard of documented return to the index hospital among survivors of the index admission and should not be interpreted as estimating complete population-level 90-day all-cause readmission.

### 2.7 Statistical analysis

Analyses were conducted according to a predefined outcome hierarchy, with the composite adverse in-hospital outcome designated as the primary outcome and documented 90-day readmission to the index hospital treated as a secondary exploratory outcome. Continuous variables were summarised as mean ± standard deviation and categorical variables as counts and percentages. Between-group comparisons used independent-samples t tests or Mann–Whitney U tests for continuous variables, and χ^2^ or Fisher’s exact tests for categorical variables, as appropriate. Two-sided *p* values <0.05 were considered statistically significant. No imputation was performed. Twenty-six admissions were excluded before final cohort assembly because information required for eligibility or endpoint ascertainment could not be recovered, whereas the covariates included in the primary multivariable models were complete in the final analytic cohorts.

To identify factors associated with the composite adverse in-hospital outcome, we first fitted a base multivariable logistic regression model including age, Charlson Comorbidity Index, Clinical Frailty Scale ≥5, peripheral oxygen saturation on room air <92%, serum albumin <35 g/L, aspiration-related LRTI, and sepsis or septic shock at admission. A hierarchical Parkinson’s disease–specific model was then constructed by adding Hoehn–Yahr stage 3–5 and pre-admission wheelchair- or bed-bound status to the base model. Covariates in the base model were selected before model fitting on clinical grounds to represent age, comorbidity burden, frailty, oxygenation, hypoalbuminaemia, aspiration phenotype, and systemic infection severity. Hoehn–Yahr stage and pre-admission wheelchair- or bed-bound status were added hierarchically because they represented complementary measures of Parkinson’s disease–related motor severity and functional dependence and had relatively complete documentation. Covariate selection was not based on univariable *p* values, automated stepwise procedures, or optimisation of model discrimination. Nested-model fit was compared using the likelihood-ratio test, and discrimination was described using the area under the receiver operating characteristic curve; these measures were used to characterise model behaviour rather than to establish a validated clinical prediction tool. Variance inflation factors were examined to assess multicollinearity. Internal validation of the base and Parkinson’s disease–specific extended logistic models was performed using 1,000 bootstrap resamples. Optimism-corrected C-statistics and bootstrap calibration slopes were calculated by estimating model performance in each bootstrap sample and comparing it with performance in the original cohort. These analyses were used to quantify model optimism and were not intended to establish clinical transportability.

Parkinson’s disease duration, tube feeding at admission, treatment with three or more antiparkinsonian medications, and prior antipsychotic use for hallucinations or psychosis were evaluated separately in additional sensitivity models because of clinical overlap among Parkinson’s disease–related severity, functional, and treatment markers and the limited number of primary outcome events. Each variable was added separately to the base model. Adjusted odds ratios with 95% confidence intervals and two-sided *p* values are reported. As a sensitivity analysis, invasive mechanical ventilation or in-hospital death was analysed as a harder in-hospital endpoint. Because 38 patients experienced this endpoint, a Firth penalised logistic regression model was fitted using the same covariates as the Parkinson’s disease–specific extended model to reduce small-sample and separation bias. These results were considered supportive of sensitivity analyses rather than a separate prediction model.

In the secondary exploratory analysis, time to first documented readmission to the index hospital within 90 days among survivors of the index admission was analysed using Cox proportional hazards regression (Table 1; Figure 1A). Follow-up time was calculated from discharge to the first documented readmission to the index hospital, with administrative censoring at 90 days for patients without a recorded same-hospital readmission. The multivariable Cox model included age, Charlson Comorbidity Index, baseline serum albumin <35 g/L, pneumonia-related hospitalisation within the previous 12 months, occurrence of the composite adverse in-hospital outcome, length of stay, discharge disposition, and aspiration-related LRTI. These covariates were selected on clinical grounds to represent baseline vulnerability, prior respiratory instability, the index hospital course, and discharge context and were not selected according to univariable statistical significance. Results are presented as adjusted hazard ratios (HRs) with 95% CIs and *p* values. Discriminative performance was assessed using Harrell’s C-index with 95% CIs, and the proportional hazards assumption was evaluated using Schoenfeld residuals. The Cox model was internally validated using 1,000 bootstrap resamples, from which an optimism-corrected C-index and bootstrap calibration slope were estimated.

**Table 1:** Exploratory cox proportional hazards analysis of documented 90-day readmission to the index hospital after hospitalisation for lower respiratory tract infection in older patients with Parkinson’s disease.

Variable or model measure	Estimate	95% CI	*p* value
Age, per 1-year increase	1.01	0.98–1.05	0.412
Charlson comorbidity index, per 1-point increase	1.13	1.01–1.27	0.033
Serum albumin <35 g/L at admission	1.82	1.12–2.95	0.016
Prior hospitalisation for pneumonia within 12 months	1.69	1.05–2.73	0.031
Composite adverse in-hospital outcome*	1.78	1.07–2.97	0.026
Length of stay, per 1-day increase	1.03	1.00–1.06	0.048
Discharge to a non-home setting†	1.96	1.19–3.24	0.008
Aspiration-related LRTI	1.43	0.88–2.32	0.147
Number of patients discharged alive	228	—	—
Number of documented readmissions	74	—	—
Apparent Harrell’s C-index	0.73	0.67–0.79	—
Optimism-corrected C-index	0.70	—	—
Bootstrap calibration slope	0.86	—	—
Number of bootstrap resamples	1,000	—	—
Global proportional hazards test	—	—	0.590

Estimates for clinical covariates are adjusted HRs. The model included eight covariates and 74 documented readmissions, corresponding to approximately 9.3 events per covariate. Internal validation was performed using 1,000 bootstrap resamples. The apparent Harrell’s C-index was 0.73, and the optimism-corrected C-index was 0.70, with a bootstrap calibration slope of 0.86. The proportional hazards assumption was evaluated using Schoenfeld residuals. Readmissions to other hospitals and post-discharge deaths were not systematically available; the HRs therefore refer to documented return to the index hospital and should not be interpreted as complete population-level 90-day all-cause readmission risk. *Composite adverse in-hospital outcome was defined as the occurrence of at least one of the following during the index hospitalisation: ICU admission, invasive mechanical ventilation, or in-hospital death. †Discharge to a non-home setting included transfer to a rehabilitation ward or hospital, nursing or long-term care facility, or another acute-care hospital.

**Figure 1:**
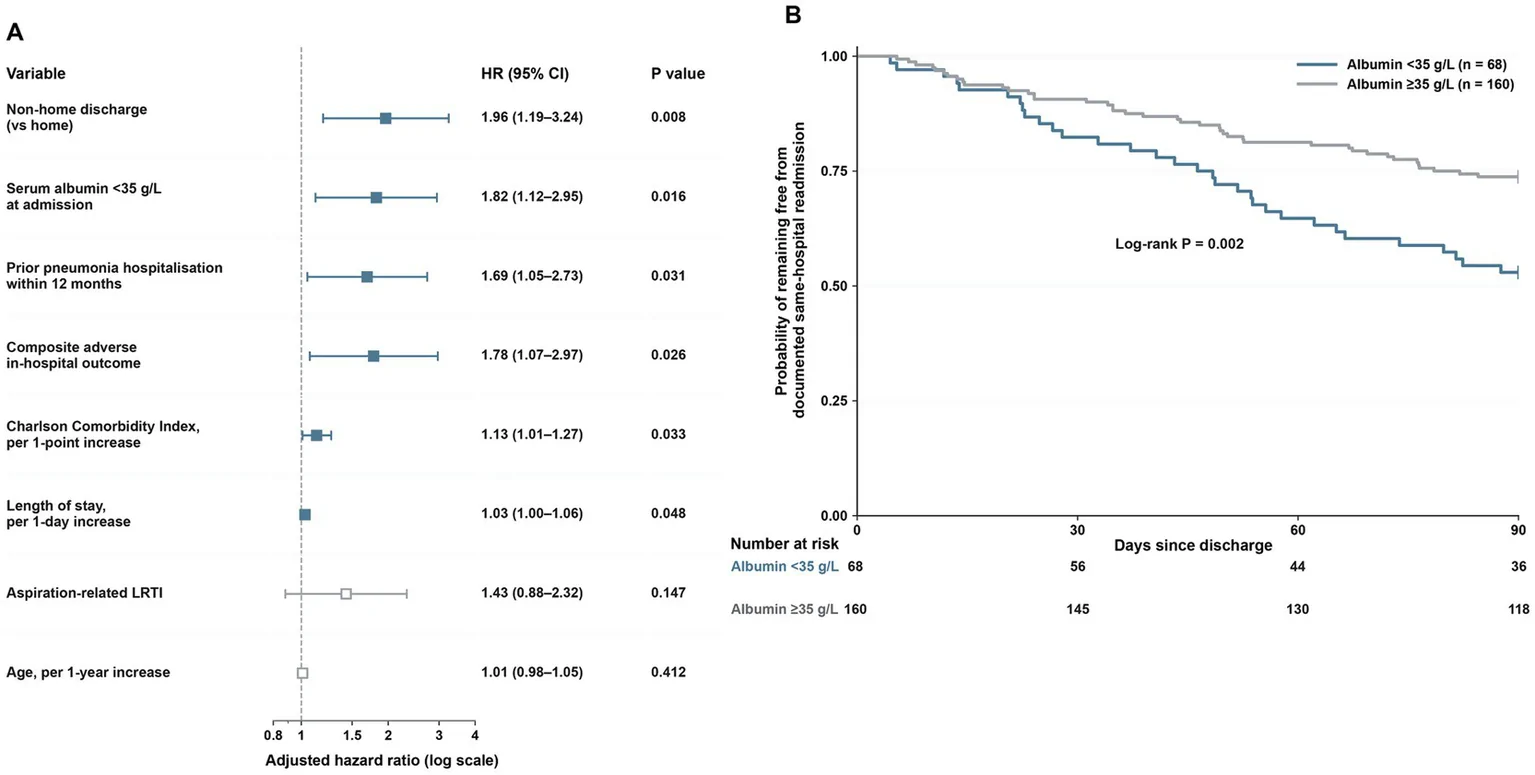
Factors associated with documented 90-day readmission to the index hospital and readmission-free probability. **(A)** Forest plot of adjusted HRs and 95% CIs from the exploratory Cox proportional hazards model among 228 patients discharged alive, of whom 74 had a documented readmission to the index hospital within 90 days. Filled blue squares indicate variables with *p* < 0.05, and open grey squares indicate variables with *p* ≥ 0.05. Apparent model discrimination and the global proportional hazards test are shown below the plot; bootstrap-corrected performance is reported in Table 1. **(B)** Kaplan–Meier curves showing the probability of remaining free from documented same-hospital readmission according to serum albumin category at admission. Numbers at risk at 0, 30, 60, and 90 days are shown below the x-axis. The curves differed by the log-rank test (*p* = 0.002).

To examine the adjusted associations of Parkinson’s disease–related characteristics with the post-discharge endpoint, Hoehn–Yahr stage 3–5, pre-admission wheelchair- or bed-bound status, Parkinson’s disease duration, tube feeding at admission, and use of three or more antiparkinsonian medications were each added separately to the exploratory Cox model. These variables were evaluated separately to limit model overfitting and reduce the influence of collinearity among related measures of disease severity, frailty, and functional dependence. Kaplan–Meier curves for remaining free from documented readmission to the index hospital were generated according to baseline albumin category (<35 vs. ≥35 g/L) and compared using the log-rank test (Figure 1B). All statistical analyses were conducted using R software (R Foundation for Statistical Computing, Vienna, Austria).

Three additional analyses were performed to evaluate potential calendar-time effects. First, patient numbers and crude outcome rates were summarised separately for admissions occurring during 2018–2019, 2020–2022, and 2023–2025. Second, the multivariable logistic and Cox regression models were repeated with calendar period included as a three-level categorical covariate, using 2018–2019 as the reference period. Third, both models were refitted after excluding admissions occurring during 2020–2022. These analyses were considered sensitivity analyses because individual-level SARS-CoV-2 infection and detailed pandemic-related changes in care were not uniformly available.

### 2.8 Ethics

The study protocol was reviewed and approved by the Clinical Trial Ethics Committee of the Affiliated Hospital of Southwest Medical University (approval no. KY2026193). Given the retrospective design and the use of existing clinical records, the requirement for written informed consent was waived by the ethics committee. Patient data were de-identified before statistical analysis and were handled in accordance with applicable institutional and national requirements for clinical research and data protection.

## 3 Results

### 3.1 Cohort assembly and baseline characteristics

Between 2018 and 2025, 382 hospitalisations involving patients with concurrent Parkinson’s disease (PD) and lower respiratory tract infection (LRTI) were identified. Forty-four admissions involving patients aged <65 years, 18 stays shorter than 24 h, 32 admissions in which LRTI was not the primary diagnosis, 16 admissions with explicitly documented end-of-life or comfort-only treatment goals, and 26 admissions with non-recoverable information required for eligibility or endpoint ascertainment were excluded. The final cohort comprised 246 patients, of whom 62 (25.2%) experienced the composite adverse in-hospital outcome, and 184 (74.8%) experienced none of its components. Eighteen patients died during the index admission, leaving 228 survivors for the secondary post-discharge analysis. Sixty admissions occurred during 2018–2019, 78 during 2020–2022, and 108 during 2023–2025. The corresponding rates of the composite outcome were 26.7, 29.5, and 21.3% (*p* = 0.427), while documented readmission to the index hospital among survivors occurred in 34.5, 34.7, and 29.7%, respectively (*p* = 0.731; Figures 2, 3A and Supplementary Table S1).

**Figure 2:**
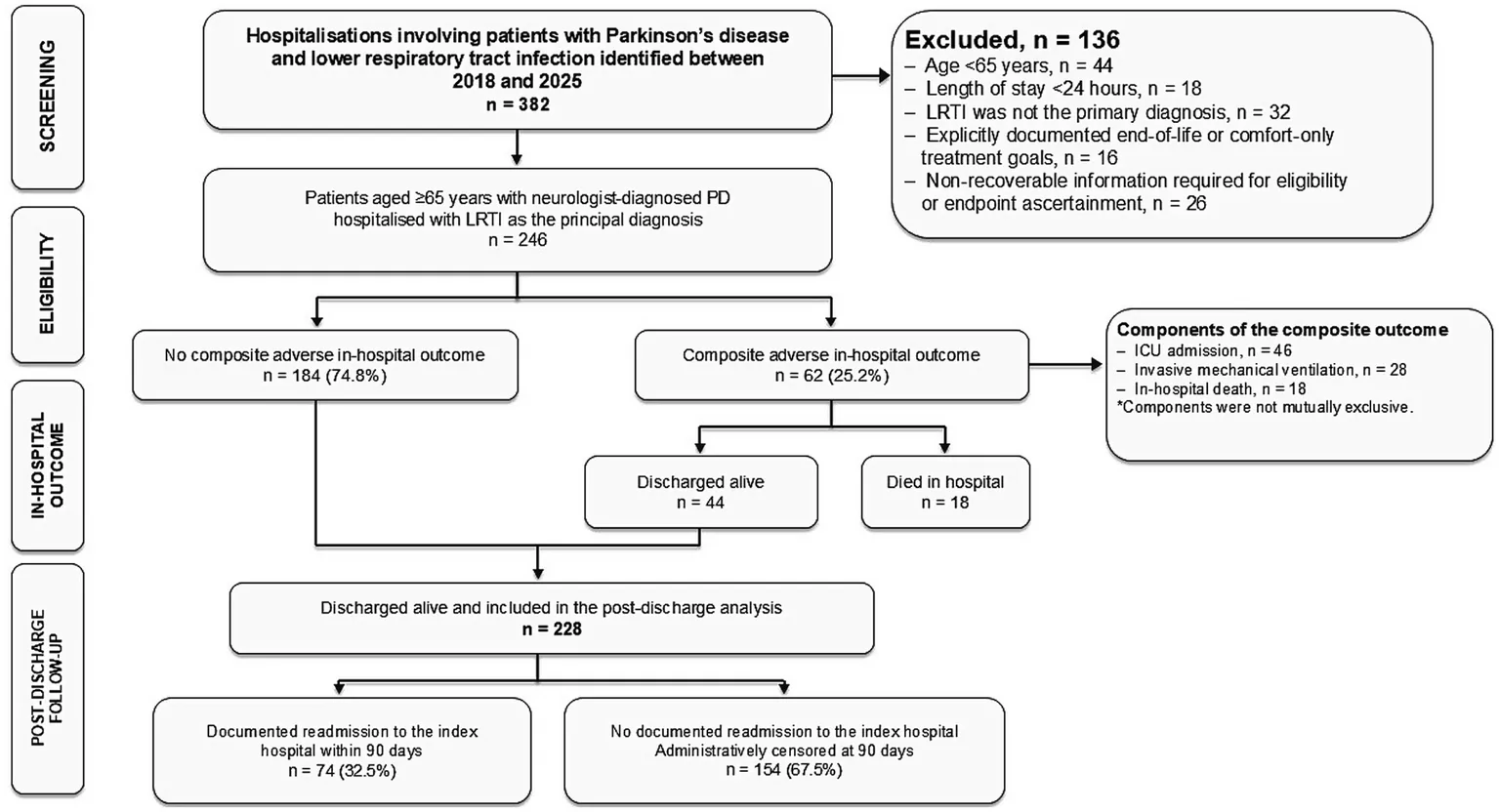
Study flow diagram of cohort selection and outcome analyses. A total of 382 hospitalisations involving patients with Parkinson’s disease and lower respiratory tract infection were screened, of which 136 were excluded according to the eligibility criteria. The final cohort comprised 246 patients; 62 (25.2%) experienced the composite adverse in-hospital outcome, and 184 (74.8%) did not. The composite outcome comprised ICU admission, invasive mechanical ventilation, or in-hospital death, and the individual components were not mutually exclusive. Eighteen patients died during the index admission, leaving 228 patients discharged alive for the secondary post-discharge analysis. Within 90 days after discharge, 74 patients had a documented readmission to the index hospital, whereas 154 had no documented same-hospital readmission and were administratively censored at 90 days.

**Figure 3:**
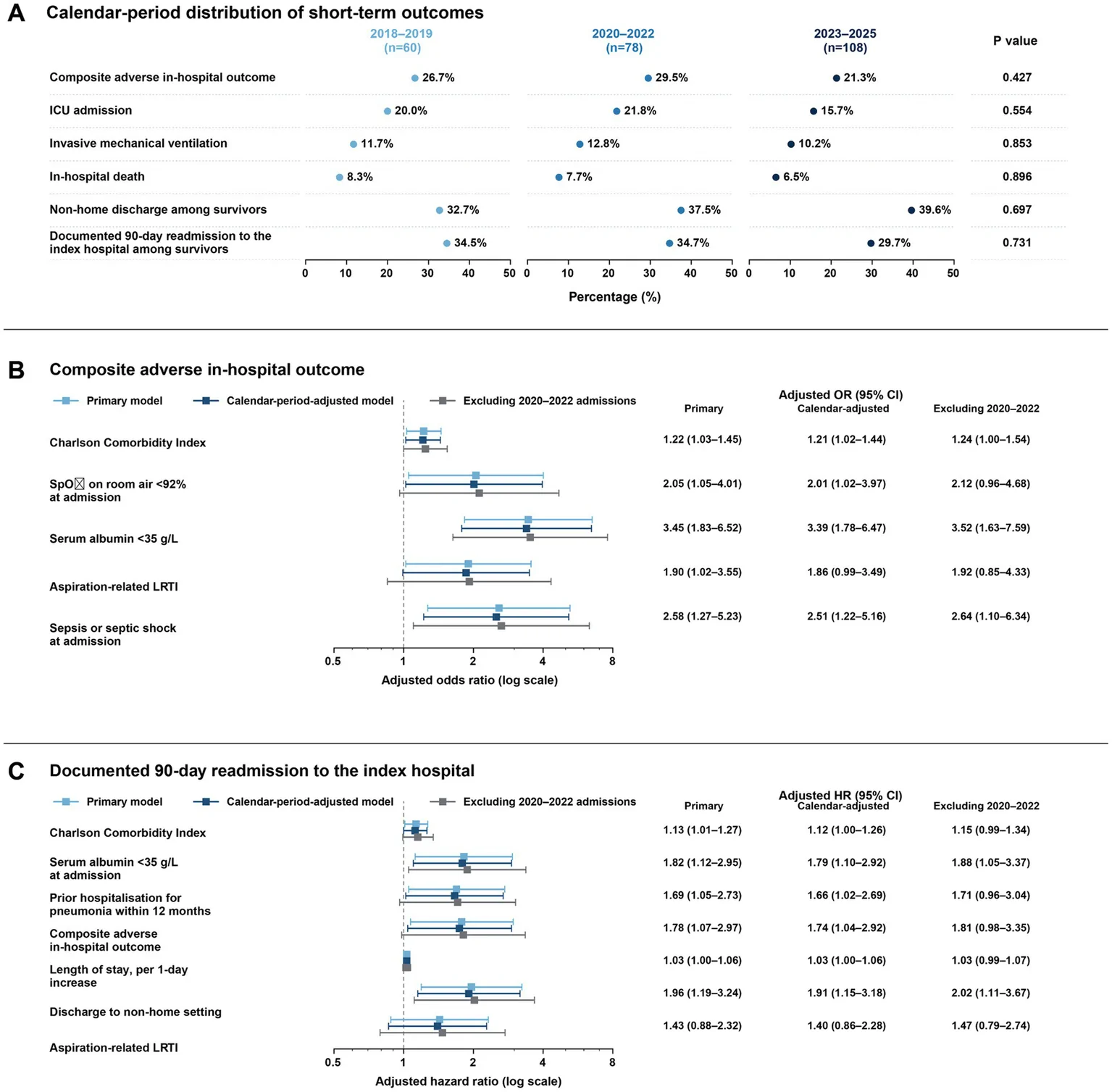
Calendar-period variation in short-term outcomes and sensitivity analyses of adjusted associations. **(A)** Crude short-term outcome rates across the 2018–2019, 2020–2022, and 2023–2025 calendar periods. **(B)** Adjusted ORs and 95% CIs from the base logistic model, the calendar-period-adjusted model, and the model excluding 2020–2022 admissions. **(C)** Adjusted HRs and 95% CIs from the exploratory Cox model using the same three analytical settings. Squares indicate point estimates, horizontal lines indicate 95% CIs, and dashed vertical lines indicate the null value of 1. Full estimates are reported in Supplementary Tables S1–S3.

Compared with patients without the composite outcome, those with adverse in-hospital outcomes were older, had longer PD duration, and more frequently had Hoehn–Yahr stage 3–5, pre-admission wheelchair- or bed-bound status, tube feeding, and residence in a nursing home or long-term care facility. They also more frequently had a hospitalisation, a pneumonia-related hospitalisation, or a fall during the preceding 12 months and were more likely to receive long-term home oxygen therapy. Comorbidity and frailty burdens were higher, with a greater Charlson Comorbidity Index (5.1 ± 1.6 vs. 4.1 ± 1.4; *p* < 0.001) and a higher prevalence of Clinical Frailty Scale scores ≥5 (61.3% vs. 39.1%; *p* = 0.003). At admission, the adverse-outcome group had lower room-air SpO₂, more pronounced inflammatory abnormalities, lower estimated glomerular filtration rate, and lower serum albumin concentrations (33.4 ± 4.5 vs. 37.9 ± 4.0 g/L; *p* < 0.001). Hypoalbuminaemia, aspiration-related LRTI, and sepsis or septic shock were also more frequent in this group (Table 2).

**Table 2:** Baseline characteristics of older patients with Parkinson’s disease hospitalised for lower respiratory tract infection, according to composite adverse in-hospital outcome.

Characteristic	No composite adverse in-hospital outcome (*n* = 184)	Composite adverse in-hospital outcome (*n* = 62)	Statistical test (value)	*p* value
Demographics and PD-related characteristics				
Age, years	76.4 ± 6.3	79.0 ± 7.0	Student t test (t = −2.52)	0.013
Male sex, *n* (%)	60.3% (111/184)	69.4% (43/62)	Chi-square test (χ^2^ = 1.70)	0.192
Body mass index, kg/m^2^	22.9 ± 3.1	21.8 ± 3.4	Student t test (t = 2.10)	0.037
Parkinson’s disease duration, years	7.0 ± 4.2	8.6 ± 4.8	Student t test (t = −2.42)	0.017
Hoehn–Yahr stage 3–5, *n* (%)	57.6% (106/184)	77.4% (48/62)	Chi-square test (χ^2^ = 7.93)	0.005
Residence in nursing home/long-term care facility, *n* (%)	9.8% (18/184)	22.6% (14/62)	Chi-square test (χ^2^ = 6.40)	0.011
Pre-admission wheelchair- or bed-bound status, *n* (%)	20.7% (38/184)	46.8% (29/62)	Chi-square test (χ^2^ = 14.61)	<0.001
Tube feeding (nasogastric or gastrostomy) at admission, *n* (%)	10.9% (20/184)	29.0% (18/62)	Chi-square test (χ^2^ = 11.15)	0.001
Lifestyle and recent clinical history				
Current or former smoker, *n* (%)	31.0% (57/184)	37.1% (23/62)	Chi-square test (χ^2^ = 0.79)	0.375
Regular alcohol consumption, *n* (%)	12.5% (23/184)	14.5% (9/62)	Chi-square test (χ^2^ = 0.15)	0.697
≥1 hospitalisation in previous 12 months, *n* (%)	42.9% (79/184)	62.9% (39/62)	Chi-square test (χ^2^ = 7.27)	0.007
Hospitalisation for pneumonia in previous 12 months, *n* (%)	21.7% (40/184)	37.1% (23/62)	Chi-square test (χ^2^ = 5.10)	0.024
≥1 fall in previous 12 months, *n* (%)	30.4% (56/184)	46.8% (29/62)	Chi-square test (χ^2^ = 5.21)	0.022
Long-term home oxygen therapy, *n* (%)	7.1% (13/184)	17.7% (11/62)	Chi-square test (χ^2^ = 5.24)	0.022
Comorbidities				
Hypertension, *n* (%)	70.1% (129/184)	79.0% (49/62)	Chi-square test (χ^2^ = 1.80)	0.180
Diabetes mellitus, *n* (%)	37.5% (69/184)	46.8% (29/62)	Chi-square test (χ^2^ = 1.71)	0.191
Coronary artery disease, *n* (%)	28.8% (53/184)	40.3% (25/62)	Chi-square test (χ^2^ = 2.68)	0.102
Chronic heart failure, *n* (%)	15.8% (29/184)	29.0% (18/62)	Chi-square test (χ^2^ = 4.97)	0.026
Atrial fibrillation, *n* (%)	11.4% (21/184)	19.4% (12/62)	Chi-square test (χ^2^ = 2.30)	0.130
Chronic obstructive pulmonary disease, *n* (%)	21.2% (39/184)	33.9% (21/62)	Chi-square test (χ^2^ = 3.90)	0.048
Chronic kidney disease, *n* (%)	13.0% (24/184)	21.0% (13/62)	Chi-square test (χ^2^ = 2.27)	0.132
Prior ischaemic or haemorrhagic stroke, *n* (%)	26.1% (48/184)	46.8% (29/62)	Chi-square test (χ^2^ = 9.12)	0.003
Dementia or cognitive impairment, *n* (%)	18.5% (34/184)	33.9% (21/62)	Chi-square test (χ^2^ = 6.57)	0.010
Depressive disorder, *n* (%)	22.8% (42/184)	25.8% (16/62)	Chi-square test (χ^2^ = 0.21)	0.644
Active malignancy, *n* (%)	9.2% (17/184)	12.9% (8/62)	Chi-square test (χ^2^ = 0.63)	0.428
Charlson comorbidity index, points	4.1 ± 1.4	5.1 ± 1.6	Student t test (t = −4.09)	<0.001
Frailty and PD treatment status				
Clinical frailty scale ≥5, *n* (%)	39.1% (72/184)	61.3% (38/62)	Chi-square test (χ^2^ = 9.01)	0.003
≥3 antiparkinsonian drugs, *n* (%)	28.3% (52/184)	37.1% (23/62)	Chi-square test (χ^2^ = 1.63)	0.202
Prior antipsychotic use for hallucinations/psychosis, *n* (%)	15.8% (29/184)	25.8% (16/62)	Chi-square test (χ^2^ = 2.94)	0.087
Vital signs at admission				
Systolic blood pressure, mmHg	133.0 ± 16.5	130.4 ± 18.1	Student t test (t = 0.97)	0.334
Heart rate, beats/min	85.3 ± 11.5	91.0 ± 13.8	Student t test (t = −3.20)	0.002
Respiratory rate, breaths/min	20.1 ± 3.0	22.0 ± 3.6	Student t test (t = −3.37)	0.001
Body temperature, °C	37.7 ± 0.8	37.9 ± 0.9	Student t test (t = −1.51)	0.133
SpO₂ on room air, %	94.3 ± 2.7	90.8 ± 4.3	Student t test (t = 6.57)	<0.001
Oxygen therapy initiated at admission, *n* (%)	46.2% (85/184)	77.4% (48/62)	Chi-square test (χ^2^ = 17.01)	<0.001
Haematology and inflammatory markers				
Haemoglobin, g/L	121.0 ± 16.0	115.8 ± 17.5	Student t test (t = 2.00)	0.048
White blood cell count, ×10^9^/L	9.7 ± 3.3	11.0 ± 3.9	Student t test (t = −2.39)	0.018
Neutrophil percentage, %	78.2 ± 9.0	82.1 ± 8.3	Student t test (t = −2.69)	0.008
Lymphocyte count, ×10^9^/L	1.3 ± 0.5	1.0 ± 0.4	Student t test (t = 3.61)	<0.001
C-reactive protein, mg/L	70.1 ± 44.0	108.5 ± 58.0	Mann–Whitney U test (Z = −3.82)	<0.001
Procalcitonin, ng/mL	0.32 ± 0.27	0.55 ± 0.43	Mann–Whitney U test (Z = −3.02)	0.003
Liver, kidney, albumin, and electrolyte status				
Alanine aminotransferase, U/L	25.0 ± 11.5	27.2 ± 13.8	Student t test (t = −1.21)	0.228
Aspartate aminotransferase, U/L	28.3 ± 12.5	33.0 ± 15.3	Student t test (t = −2.08)	0.038
Serum creatinine, μmol/L	83.5 ± 26.0	95.0 ± 34.0	Student t test (t = −2.40)	0.018
eGFR, mL/min/1.73 m^2^	72.6 ± 18.0	61.7 ± 20.0	Student t test (t = 3.39)	0.001
Serum albumin, g/L	37.9 ± 4.0	33.4 ± 4.5	Student t test (t = 7.12)	<0.001
Serum albumin <35 g/L, *n* (%)	22.3% (41/184)	62.9% (39/62)	Chi-square test (χ^2^ = 33.64)	<0.001
Serum sodium, mmol/L	138.1 ± 3.5	137.3 ± 4.0	Student t test (t = 1.40)	0.163
Serum potassium, mmol/L	4.0 ± 0.4	4.1 ± 0.5	Student t test (t = −1.09)	0.276
Current LRTI-related characteristics				
Aspiration-related LRTI, *n* (%)	22.8% (42/184)	40.3% (25/62)	Chi-square test (χ^2^ = 6.96)	0.008
Healthcare-associated pneumonia, *n* (%)	18.5% (34/184)	27.4% (17/62)	Chi-square test (χ^2^ = 2.01)	0.156
Sepsis or septic shock at admission, *n* (%)	11.4% (21/184)	32.3% (20/62)	Chi-square test (χ^2^ = 14.62)	<0.001

Values are mean ± SD or *n* (%) as indicated. PD, Parkinson’s disease; LRTI, lower respiratory tract infection; eGFR, estimated glomerular filtration rate; CFS, Clinical Frailty Scale; ICU, intensive care unit.

### 3.2 In-hospital management, complications, and clinical outcomes

Patients who experienced the composite outcome received more intensive in-hospital treatment (Table 3). They received the first antibiotic earlier and were more frequently treated with carbapenems, empirical combinations involving at least two antibiotic classes, and regimens providing anti-Pseudomonas or anaerobic coverage. Intravenous and total antibiotic treatment durations were also longer. Respiratory and haemodynamic support was more common, including oxygen therapy (96.8% vs. 68.5%), non-invasive ventilation (51.6% vs. 21.7%), and vasopressor use (32.3% vs. 3.3%; all *p* < 0.001). Enteral or parenteral nutrition, formal nutritional assessment, and swallowing or rehabilitation consultation were used more frequently, whereas mobilisation within 48 h was less common (21.0% vs. 44.0%; *p* = 0.001).

**Table 3:** In-hospital management, complications, and outcomes of older patients with Parkinson’s disease hospitalised for lower respiratory tract infection, according to a composite adverse in-hospital outcome.

Variable	No composite adverse in-hospital outcome (*n* = 184)	Composite adverse in-hospital outcome (*n* = 62)	Statistical test (value)	*p* value
General information				
Age, years	76.4 ± 6.3	79.0 ± 7.0	Student t test (t = −2.52)	0.013
Male sex, *n* (%)	60.3% (111/184)	69.4% (43/62)	Chi-square test (χ^2^ = 1.70)	0.192
Antimicrobial therapy				
Time from admission to first antibiotic, hours	6.8 ± 4.2	5.4 ± 3.6	Mann–Whitney U test (Z = −2.02)	0.043
Initial regimen: β-lactam/β-lactamase inhibitor, *n* (%)	42.4% (78/184)	29.0% (18/62)	Chi-square test (χ^2^ = 3.76)	0.053
Initial regimen: third-generation cephalosporin ± macrolide, *n* (%)	30.4% (56/184)	17.7% (11/62)	Chi-square test (χ^2^ = 3.42)	0.064
Initial regimen: respiratory fluoroquinolone, *n* (%)	15.8% (29/184)	8.1% (5/62)	Chi-square test (χ^2^ = 2.13)	0.144
Initial regimen: carbapenem, *n* (%)	3.3% (6/184)	12.9% (8/62)	Chi-square test (χ^2^ = 8.20)	0.004
Initial combination regimen (≥2 antibiotic classes), *n* (%)	8.2% (15/184)	30.6% (19/62)	Chi-square test (χ^2^ = 19.77)	<0.001
Anti-Pseudomonas coverage, *n* (%)	26.1% (48/184)	53.2% (33/62)	Chi-square test (χ^2^ = 15.26)	<0.001
Anaerobic coverage, *n* (%)	30.4% (56/184)	46.8% (29/62)	Chi-square test (χ^2^ = 5.21)	0.022
Duration of intravenous antibiotics, days	8.2 ± 3.1	10.5 ± 4.0	Mann–Whitney U test (Z = −3.47)	0.001
Total duration of antibiotic therapy, days	10.7 ± 3.8	13.2 ± 4.5	Mann–Whitney U test (Z = −3.62)	<0.001
Respiratory and circulatory support				
Any oxygen therapy during hospital stay, *n* (%)	68.5% (126/184)	96.8% (60/62)	Chi-square test (χ^2^ = 22.64)	<0.001
Non-invasive ventilation, *n* (%)	21.7% (40/184)	51.6% (32/62)	Chi-square test (χ^2^ = 18.11)	<0.001
Invasive mechanical ventilation, *n* (%)	0.0% (0/184)	45.2% (28/62)	Chi-square test (χ^2^ = 96.72)	<0.001
ICU admission, *n* (%)	0.0% (0/184)	74.2% (46/62)	Chi-square test (χ^2^ = 147.65)	<0.001
Vasopressor use, *n* (%)	3.3% (6/184)	32.3% (20/62)	Chi-square test (χ^2^ = 28.06)	<0.001
Nutritional and rehabilitation management				
Oral nutritional supplements, *n* (%)	30.4% (56/184)	51.6% (32/62)	Chi-square test (χ^2^ = 8.41)	0.004
Enteral nutrition via tube (nasogastric/gastrostomy), *n* (%)	21.2% (39/184)	53.2% (33/62)	Chi-square test (χ^2^ = 23.09)	<0.001
Parenteral nutrition, *n* (%)	13.6% (25/184)	32.3% (20/62)	Chi-square test (χ^2^ = 10.13)	0.001
Nutrition consultation, *n* (%)	19.6% (36/184)	38.7% (24/62)	Chi-square test (χ^2^ = 8.17)	0.004
Swallowing or rehabilitation consultation, *n* (%)	27.2% (50/184)	51.6% (32/62)	Chi-square test (χ^2^ = 11.47)	0.001
Early mobilisation within 48 h (bed-to-chair/standing), *n* (%)	44.0% (81/184)	21.0% (13/62)	Chi-square test (χ^2^ = 10.81)	0.001
In-hospital complications				
Delirium, *n* (%)	15.8% (29/184)	43.5% (27/62)	Chi-square test (χ^2^ = 19.56)	<0.001
Acute kidney injury, *n* (%)	8.2% (15/184)	27.4% (17/62)	Chi-square test (χ^2^ = 14.31)	<0.001
Hospital-acquired infection (e.g., HAP/VAP, catheter-related), *n* (%)	11.4% (21/184)	29.0% (18/62)	Chi-square test (χ^2^ = 10.61)	0.001
In-hospital falls, *n* (%)	3.8% (7/184)	9.7% (6/62)	Chi-square test (χ^2^ = 3.06)	0.080
Pressure ulcers, *n* (%)	4.9% (9/184)	14.5% (9/62)	Chi-square test (χ^2^ = 6.02)	0.014
Venous thromboembolism, *n* (%)	2.2% (4/184)	6.5% (4/62)	Chi-square test (χ^2^ = 2.40)	0.121
In-hospital outcomes				
Length of hospital stay, days	10.5 ± 4.2	15.8 ± 7.0	Mann–Whitney U test (Z = −5.01)	<0.001
Composite adverse in-hospital outcome*, *n* (%)	0.0% (0/184)	100.0% (62/62)	—	—
In-hospital death, *n* (%)	0.0% (0/184)	29.0% (18/62)	Chi-square test (χ^2^ = 63.71)	<0.001
Discharge disposition among survivors†	(*n* = 184)	(*n* = 44)		
Discharged home, *n* (%)	69.6% (128/184)	34.1% (15/44)	Chi-square test (χ^2^ = 18.39)	<0.001
Transferred to rehabilitation ward/hospital, *n* (%)	17.9% (33/184)	31.8% (14/44)	Chi-square test (χ^2^ = 4.11)	0.043
Discharged to nursing facility/long-term care, *n* (%)	7.1% (13/184)	20.5% (9/44)	Chi-square test (χ^2^ = 7.12)	0.008
Transferred to another acute-care hospital, *n* (%)	5.4% (10/184)	13.6% (6/44)	Chi-square test (χ^2^ = 3.82)	0.051

Values are mean ± SD or *n* (%) as indicated. *Composite adverse in-hospital outcome was defined as the occurrence of at least one of the following during the index hospitalisation: ICU admission, invasive mechanical ventilation, or in-hospital death. The individual components were not mutually exclusive. By definition, the composite endpoint was absent in the “no composite adverse outcome” group and present in all patients in the “composite adverse outcome” group. †Proportions for discharge disposition are calculated among patients discharged alive (*n* = 184 and *n* = 44, respectively). PD, Parkinson’s disease; LRTI, lower respiratory tract infection; ICU, intensive care unit; HAP, hospital-acquired pneumonia; VAP, ventilator-associated pneumonia.

Delirium, acute kidney injury, hospital-acquired infection, and pressure ulcers occurred more frequently among patients with the composite outcome, and their hospital stay was longer (15.8 ± 7.0 vs. 10.5 ± 4.2 days; *p* < 0.001). Among survivors, patients who had experienced the composite outcome were less frequently discharged home (34.1% vs. 69.6%; *p* < 0.001) and were more frequently transferred to rehabilitation or long-term care. The components of the composite endpoint were not mutually exclusive: 46 patients (18.7%) were admitted to the intensive care unit, 28 (11.4%) received invasive mechanical ventilation, and 18 (7.3%) died during the index admission. Thirty-eight patients (15.4%) experienced the harder sensitivity endpoint of invasive mechanical ventilation or in-hospital death.

### 3.3 Factors associated with composite adverse in-hospital outcomes

In the base multivariable logistic regression model, higher Charlson Comorbidity Index (adjusted OR per 1-point increase 1.22, 95% CI 1.03–1.45; *p* = 0.021), room-air SpO₂ < 92% at admission (OR 2.05, 95% CI 1.05–4.01; *p* = 0.036), serum albumin <35 g/L (OR 3.45, 95% CI 1.83–6.52; *p* < 0.001), aspiration-related LRTI (OR 1.90, 95% CI 1.02–3.55; *p* = 0.041), and sepsis or septic shock (OR 2.58, 95% CI 1.27–5.23; *p* = 0.009) were associated with the composite outcome. After Hoehn–Yahr stage 3–5 and pre-admission wheelchair- or bed-bound status were added, comorbidity burden, hypoxaemia, hypoalbuminaemia, and sepsis or septic shock retained adjusted associations. Pre-admission wheelchair- or bed-bound status was also associated with the outcome (OR 2.08, 95% CI 1.08–4.01; *p* = 0.029), whereas Hoehn–Yahr stage 3–5 was not (OR 1.59, 95% CI 0.81–3.13; *p* = 0.178). The association with aspiration-related LRTI was attenuated in the extended model (OR 1.75, 95% CI 0.91–3.36; *p* = 0.093; Table 4 and Figure 4).

**Table 4:** Hierarchical multivariable logistic regression analysis of composite adverse in-hospital outcomes in older patients with Parkinson’s disease and lower respiratory tract infection.

Variable	Base model, adjusted OR (95% CI)	*p* value	PD-specific extended model, adjusted OR (95% CI)	*p* value
Age, per 1-year increase	1.04 (0.99–1.10)	0.089	1.03 (0.98–1.09)	0.206
Charlson comorbidity index, per 1-point increase	1.22 (1.03–1.45)	0.021	1.19 (1.00–1.42)	0.049
Clinical frailty scale ≥5	1.58 (0.84–2.96)	0.153	1.29 (0.65–2.56)	0.466
SpO₂ on room air <92% at admission	2.05 (1.05–4.01)	0.036	2.00 (1.01–3.96)	0.047
Serum albumin <35 g/L	3.45 (1.83–6.52)	<0.001	3.18 (1.65–6.14)	0.001
Aspiration-related LRTI	1.90 (1.02–3.55)	0.041	1.75 (0.91–3.36)	0.093
Sepsis or septic shock at admission	2.58 (1.27–5.23)	0.009	2.41 (1.17–4.98)	0.017
Hoehn–Yahr stage 3–5	Not included	—	1.59 (0.81–3.13)	0.178
Pre-admission wheelchair- or bed-bound status	Not included	—	2.08 (1.08–4.01)	0.029
Number of patients	246	—	246	—
Number of composite events	62	—	62	—
Apparent C-statistic (95% CI)	0.82 (0.76–0.87)	—	0.84 (0.79–0.89)	—
Optimism-corrected C-statistic	0.80	—	0.81	—
Bootstrap calibration slope	0.91	—	0.87	—
Number of bootstrap resamples	1,000	—	1,000	—
Likelihood-ratio test versus base model	Reference	—	—	0.039

The base model included seven general vulnerability and acute infection-related covariates, corresponding to approximately 8.9 composite events per covariate. The PD-specific extended model additionally included Hoehn–Yahr stage and pre-admission wheelchair- or bed-bound status, corresponding to approximately 6.9 events per covariate. Variance inflation factors were <2.5 for all covariates in the extended model. Internal validation was performed using 1,000 bootstrap resamples. The optimism-corrected C-statistics were 0.80 for the base model and 0.81 for the extended model, with corresponding calibration slopes of 0.91 and 0.87. These models were developed to examine adjusted associations and compare nested model specifications rather than to provide a validated clinical prediction tool. OR, odds ratio; CI, confidence interval; PD, Parkinson’s disease; LRTI, lower respiratory tract infection; SpO₂, peripheral oxygen saturation.

**Figure 4:**
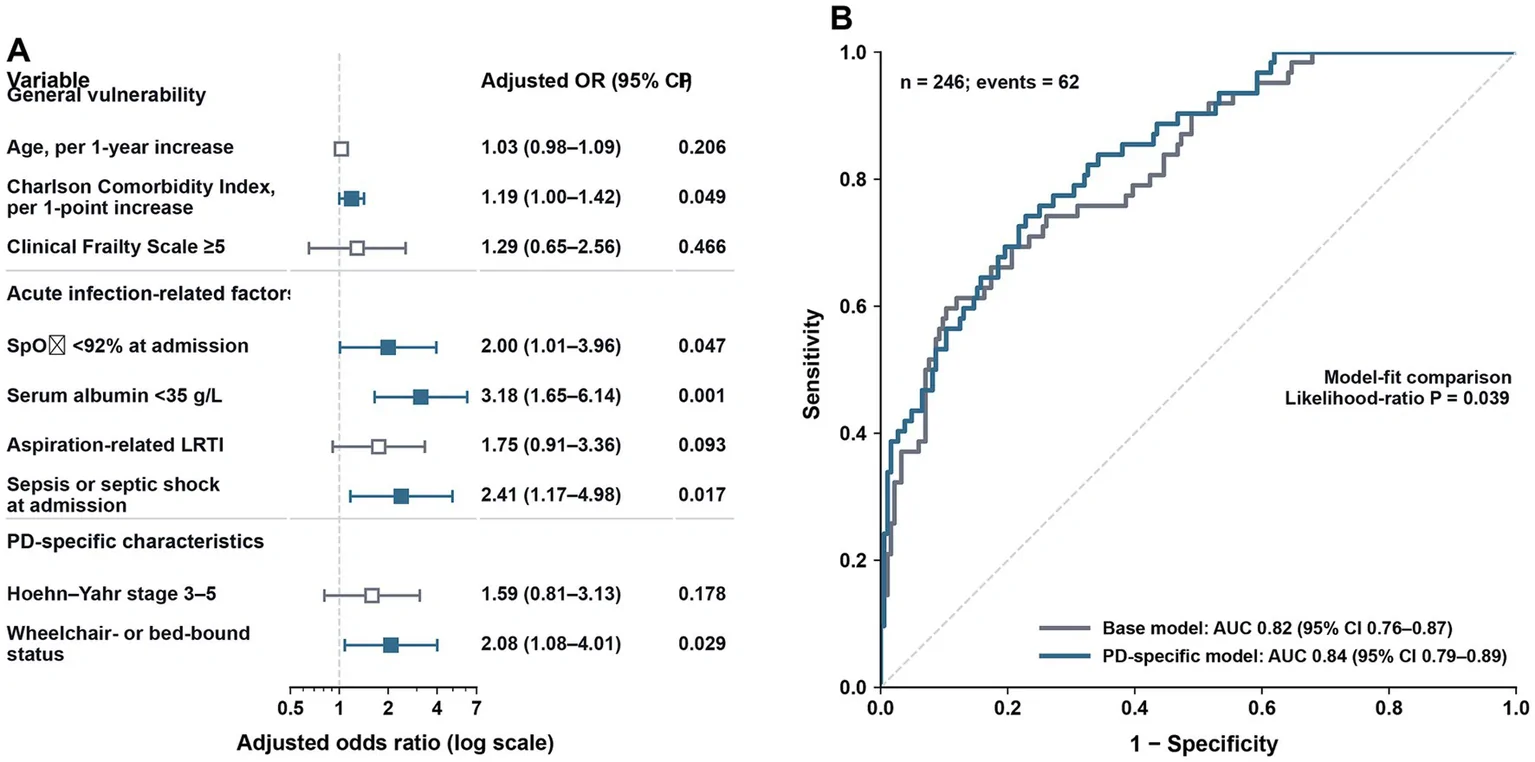
Adjusted associations and model comparison after inclusion of Parkinson’s disease–specific characteristics for composite adverse in-hospital outcomes. **(A)** Forest plot of adjusted odds ratios and 95% CIs from the Parkinson’s disease–specific extended logistic regression model among 246 patients with 62 composite adverse in-hospital events. The model included age, Charlson Comorbidity Index, Clinical Frailty Scale ≥5, admission SpO₂ < 92%, serum albumin <35 g/L, aspiration-related LRTI, sepsis or septic shock, Hoehn–Yahr stage 3–5, and pre-admission wheelchair- or bed-bound status. Filled blue squares indicate variables with *p* < 0.05, and open grey squares indicate variables with *p* ≥ 0.05. **(B)** Receiver operating characteristic curves for the base clinical model and the Parkinson’s disease–specific extended model. The apparent area under the curve was 0.82 (95% CI 0.76–0.87) for the base model and 0.84 (95% CI 0.79–0.89) for the extended model; the corresponding optimism-corrected values after 1,000 bootstrap resamples were 0.80 and 0.81. The likelihood-ratio test comparing the PD-specific extended model with the base model yielded *p* = 0.039. The difference in apparent area under the curve is presented descriptively and was not separately tested; after bootstrap correction, the C-statistics were 0.80 and 0.81, indicating limited incremental discrimination.

The apparent C-statistic was 0.82 (95% CI 0.76–0.87) in the base model and 0.84 (95% CI 0.79–0.89) in the PD-specific extended model; the likelihood-ratio test comparing the nested models yielded *p* = 0.039. After 1,000-resample bootstrap validation, the optimism-corrected C-statistics were 0.80 and 0.81, with calibration slopes of 0.91 and 0.87, respectively. In separate sensitivity models, PD duration, tube feeding at admission, treatment with three or more antiparkinsonian medications, and prior antipsychotic use for hallucinations or psychosis were not independently associated with the composite outcome. Calendar-period adjustment did not materially alter the overall pattern of associations, and analyses excluding admissions occurring during 2020–2022 yielded directionally similar estimates with wider confidence intervals (Figure 3B; Table 4; Supplementary Tables S2, S4).

In the Firth penalised logistic regression analysis of invasive mechanical ventilation or in-hospital death, higher Charlson Comorbidity Index (OR 1.21, 95% CI 1.00–1.46; *p* = 0.047), room-air SpO₂ < 92% (OR 2.28, 95% CI 1.06–4.92; *p* = 0.035), serum albumin <35 g/L (OR 3.54, 95% CI 1.65–7.57; *p* = 0.001), and sepsis or septic shock (OR 3.02, 95% CI 1.33–6.86; *p* = 0.008) remained associated with the harder clinical endpoint. The direction of associations was broadly consistent with the primary composite outcome, although estimates for several variables became less precise because of the smaller number of events (Figure 3C; Figure 5; Supplementary Table S6).

**Figure 5:**
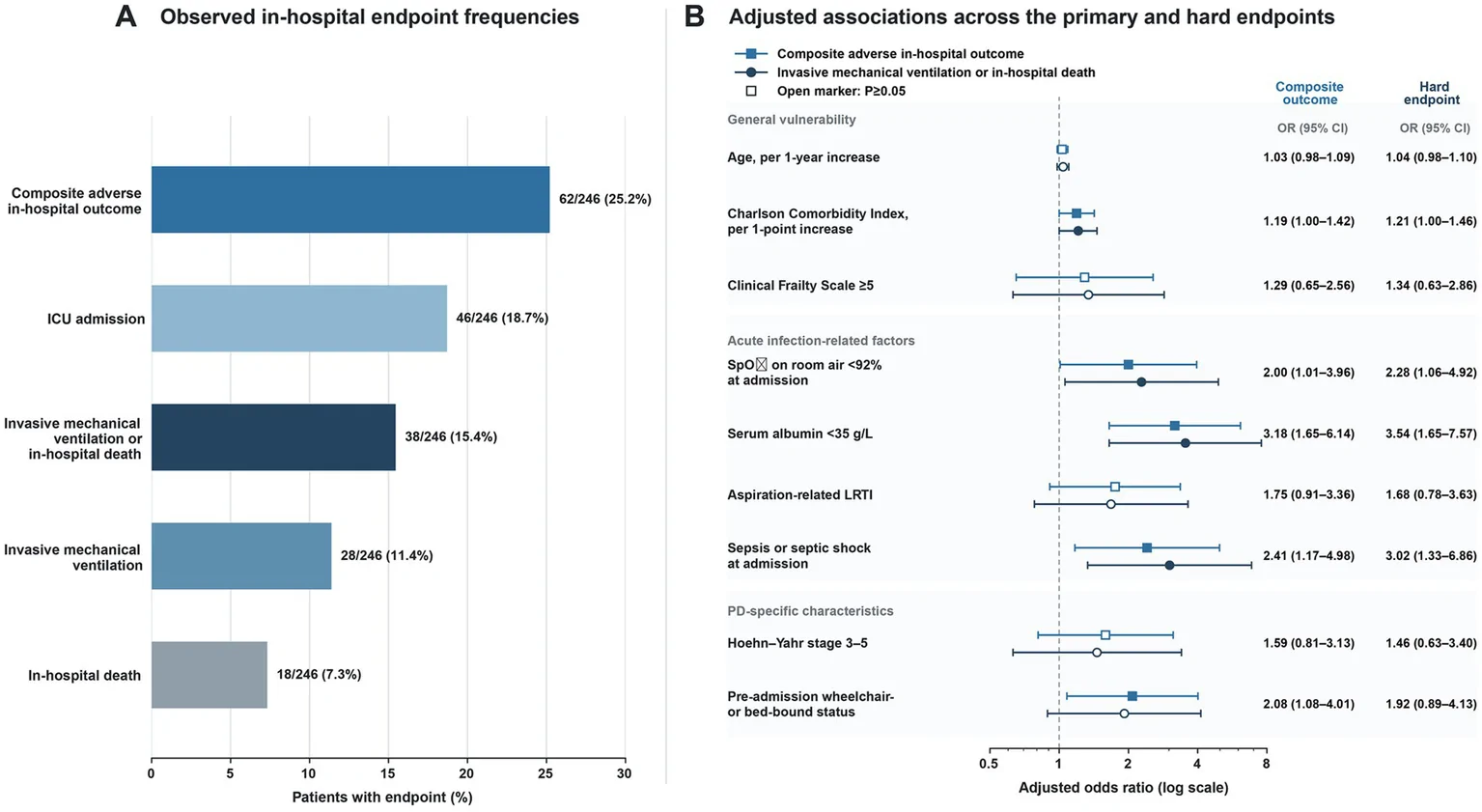
In-hospital outcome frequencies and sensitivity analysis using a harder clinical endpoint. **(A)** Frequencies of the composite adverse in-hospital outcome, its individual components, and the harder clinical endpoint among 246 patients. The harder endpoint comprised invasive mechanical ventilation or in-hospital death; individual events were not mutually exclusive **(B)** Adjusted ORs and 95% CIs for the composite outcome from the Parkinson’s disease–specific extended logistic model and for the harder endpoint from the Firth penalised logistic model using the same covariates. Filled markers indicate *p* < 0.05, open markers indicate *p* ≥ 0.05, and the dashed line indicates an OR of 1. Comparisons between endpoints are descriptive.

### 3.4 Secondary exploratory analysis of documented 90-day readmission to the index hospital

Among the 228 patients discharged alive, 74 (32.5%) had a documented readmission to the index hospital within 90 days. In the exploratory Cox model, a higher Charlson Comorbidity Index was associated with documented same-hospital readmission (adjusted HR per 1-point increase 1.13, 95% CI 1.01–1.27; *p* = 0.033). Associations were also observed for serum albumin <35 g/L (HR 1.82, 95% CI 1.12–2.95; *p* = 0.016), pneumonia-related hospitalisation within the preceding 12 months (HR 1.69, 95% CI 1.05–2.73; *p* = 0.031), the composite adverse in-hospital outcome (HR 1.78, 95% CI 1.07–2.97; *p* = 0.026), longer length of stay (HR per day 1.03, 95% CI 1.00–1.06; *p* = 0.048), and discharge to a non-home setting (HR 1.96, 95% CI 1.19–3.24; *p* = 0.008). Age and aspiration-related LRTI were not independently associated with this endpoint (Table 1; Figure 1A).

The apparent Harrell’s C-index was 0.73 (95% CI 0.67–0.79); after bootstrap validation, the optimism-corrected C-index was 0.70, and the calibration slope was 0.86. No important violation of the proportional hazards assumption was detected (global test *p* = 0.59). Calendar-period analyses produced only modest changes in the estimates (Figure 3C; Supplementary Table S3). Hoehn–Yahr stage, pre-admission wheelchair- or bed-bound status, PD duration, tube feeding, and use of three or more antiparkinsonian medications were not clearly associated with documented same-hospital readmission when added separately to the model. Kaplan–Meier analysis showed a lower probability of remaining free from documented same-hospital readmission among patients with serum albumin <35 g/L than among those with albumin ≥35 g/L (log-rank *p* = 0.002; Figure 1B; Supplementary Tables S3, S5). Because readmissions to other hospitals and post-discharge deaths were unavailable, these findings represent documented return to the index hospital rather than complete population-level all-cause readmission.

## 4 Discussion

In this single-centre cohort of older adults with Parkinson’s disease hospitalised for lower respiratory tract infection, approximately one quarter experienced intensive care unit admission, invasive mechanical ventilation, or in-hospital death. Severe in-hospital deterioration was associated with a combination of general comorbidity burden, hypoxaemia, hypoalbuminaemia, acute systemic infection severity, and pre-admission mobility dependence. Pre-admission wheelchair- or bed-bound status remained associated with the composite outcome after adjustment for general vulnerability and acute illness severity, whereas Hoehn–Yahr stage, Parkinson’s disease duration, tube feeding, and antiparkinsonian medication burden did not show clear independent associations. Among patients discharged alive, documented return to the index hospital was associated mainly with comorbidity burden, hypoalbuminaemia, previous pneumonia-related hospitalisation, a complicated index admission, longer length of stay, and non-home discharge. Internal validation indicated modest model optimism, with moderate corrected discrimination.

These findings place Parkinson’s disease–related disability within the broader context of physiological vulnerability and acute respiratory illness. Comorbidity, hypoxaemia, sepsis, frailty, and hypoalbuminaemia are recognised features of adverse outcomes in older patients with respiratory infection, while respiratory complications in Parkinson’s disease are frequently linked to swallowing dysfunction, aspiration, impaired airway clearance, and progressive functional dependence (14, 15). The present study integrates these general and disease-related domains within the same clinically defined cohort. The principal contribution of this study is therefore the within-cohort comparison of general vulnerability, acute illness severity, and Parkinson’s disease–related measures: most available Parkinson’s disease–specific measures did not retain clear independent associations after adjustment, whereas pre-admission mobility dependence did. These findings are compatible with the use of general geriatric and acute-illness assessment principles in patients with Parkinson’s disease; however, because no existing risk score was formally evaluated, the present study cannot recommend or validate any specific risk-assessment tool for this population.

General physiological vulnerability showed a consistent relationship with both the in-hospital and post-discharge endpoints. A higher Charlson Comorbidity Index may reflect reduced capacity to tolerate the respiratory, haemodynamic, renal, and metabolic consequences of acute infection. Hypoxaemia and sepsis or septic shock represent more immediate manifestations of acute physiological decompensation and remain associated with severe in-hospital deterioration after adjustment (16). Hypoalbuminaemia was also associated with both study endpoints. However, serum albumin during acute infection is influenced by systemic inflammation, capillary permeability, fluid distribution, hepatic synthetic function, chronic organ disease, and nutritional state. Albumin <35 g/L should therefore be interpreted as a nonspecific marker of limited physiological reserve and acute systemic illness rather than as a direct measure of malnutrition or an indication that albumin correction would improve outcomes.

The Parkinson’s disease–specific analyses suggest that directly observed functional dependence may be more informative during an acute respiratory admission than disease duration or treatment complexity (3, 15). Pre-admission wheelchair- or bed-bound status remained associated with the composite outcome, whereas Hoehn–Yahr stage, disease duration, tube feeding, and the number of antiparkinsonian medications did not. Mobility dependence may capture several clinically relevant consequences of advanced disease, including reduced respiratory reserve, impaired airway clearance, sarcopenia, dependence in activities of daily living, and vulnerability to hospital-associated deconditioning (17). By contrast, disease duration and medication count may correspond only loosely to current functional impairment. Because mobility status, Hoehn–Yahr stage, and frailty represent related constructs, the observed association should be interpreted as an adjusted association rather than evidence of a causal effect of immobility.

Aspiration-related LRTI was associated with the primary outcome in the base model, but the estimate was attenuated after Hoehn–Yahr stage and mobility dependence were added. This pattern may reflect overlap between aspiration phenotype, bulbar dysfunction, frailty, and advanced functional disability (1). It may also reflect the limitations of retrospective phenotype classification despite the use of clinical documentation and compatible imaging findings. In contrast, sepsis or septic shock showed a more consistent association across the hierarchical analyses, suggesting that the degree of acute systemic decompensation was more closely related to severe deterioration than the recorded aspiration phenotype alone. The sensitivity analysis using invasive mechanical ventilation or in-hospital death produced a broadly similar directional pattern for comorbidity, hypoxaemia, hypoalbuminaemia, and sepsis, although the smaller number of hard-endpoint events reduced statistical precision (Figure 5).

The post-discharge findings reflected both pre-existing vulnerability and the course of the index admission (17). Previous pneumonia-related hospitalisation may identify patients with recurrent respiratory instability, chronic aspiration, incomplete functional recovery, or repeated exposure to healthcare environments (18). The association with the composite in-hospital outcome suggests that patients experiencing severe acute deterioration may leave the hospital with greater residual physiological and functional impairment. Longer length of stay probably acts as a marker of illness complexity, complications, delayed recovery, and deconditioning rather than as a direct cause of subsequent readmission.

Non-home discharge may similarly reflect residual illness, reduced functional independence, caregiver limitations, or new rehabilitation and long-term care needs. Its association with documented return to the index hospital may also be influenced by referral agreements, transfer pathways, and thresholds for returning patients from rehabilitation or long-term care facilities. Accordingly, the post-discharge analysis characterises documented same-hospital return rather than complete population-level readmission risk. Readmissions to other hospitals and post-discharge deaths were unavailable, and patients without a recorded return were administratively censored at 90 days.

The findings support consideration of general geriatric vulnerability and acute illness severity alongside Parkinson’s disease–related functional status during hospital assessment. Comorbidity burden, oxygenation, serum albumin, systemic infection severity, and usual pre-admission mobility are readily available descriptors of clinical vulnerability, but the present analysis does not establish thresholds for risk stratification or validate a clinical decision tool. Hypoalbuminaemia may warrant contextual assessment of inflammatory burden, hydration, chronic disease, and nutritional risk, while suspected aspiration may warrant evaluation of swallowing and airway protection. The index hospital course and discharge destination may also be relevant to transitional-care planning. These observational associations do not establish that albumin supplementation, nutritional intervention, swallowing therapy, or a specific discharge programme would reduce the studied outcomes.

This study examined a clinically important population using detailed routinely collected information spanning baseline function, Parkinson’s disease severity, frailty, infection phenotype, laboratory findings, organ support, complications, and discharge disposition. The analytical structure distinguished the primary in-hospital outcome from the exploratory post-discharge endpoint and evaluated Parkinson’s disease–specific variables hierarchically. Calendar-period analyses, a harder in-hospital endpoint, separate Parkinson’s disease–related sensitivity analyses, and bootstrap internal validation were used to examine the principal findings under complementary analytical specifications.

Several limitations should be considered. The retrospective single-centre design limits causal inference and generalisability, and unmeasured differences in admission thresholds, treatment escalation, rehabilitation access, and discharge pathways may have affected the results. The study period included pre-pandemic, pandemic, and subsequent years. Although calendar-period analyses did not materially alter the overall pattern of associations, individual SARS-CoV-2 infection, caregiver availability, visitor restrictions, and pandemic-related changes in healthcare delivery could not be reconstructed consistently. The absence of a non-Parkinson’s disease comparator means that this study cannot determine whether the observed associations are specific to Parkinson’s disease or instead reflect risk patterns shared by older adults hospitalised with LRTI. ICU admission and ventilatory escalation may also have been influenced by institutional practice, treatment goals, and clinician–family decision-making. Post-discharge ascertainment was restricted to inpatient admissions recorded at the index hospital; readmissions elsewhere and deaths after discharge were unavailable, precluding complete readmission ascertainment and competing-risk analysis.

Exclusion of admissions with non-recoverable source-record incompleteness may have introduced selection bias. Excluding patients with explicitly documented end-of-life or comfort-only treatment goals may also have underrepresented those with the most advanced illness and underestimated the overall mortality burden. The number of outcome events constrained model complexity and the precision of individual estimates, and bootstrap validation does not substitute for external validation. Several clinically relevant variables, including levodopa-equivalent daily dose, motor complications, interruptions of antiparkinsonian therapy during hospitalisation, detailed motor and non-motor assessments, cognitive performance, instrumented swallowing evaluation, and formal treatment-limitation status, were incompletely documented. Hoehn–Yahr stage, mobility, frailty, aspiration-related LRTI, and sepsis were derived from routine clinical records without independent adjudication or formal inter-rater reliability assessment. Serum albumin was measured at a single time point, and validated nutritional assessments, serial albumin values, detailed fluid-balance data, and comprehensive hepatic synthetic markers were not uniformly available.

Overall, short-term outcomes in older patients with Parkinson’s disease hospitalised for LRTI were associated with both general physiological vulnerability and the severity of the acute respiratory illness. Pre-admission mobility dependence remained associated with severe in-hospital deterioration after adjustment, whereas the other available Parkinson’s disease–specific measures did not show clear independent associations. Documented return to the index hospital was related primarily to general vulnerability, the index hospital course, and discharge context. Prospective multicentre studies with standardised phenotyping, appropriate comparison groups, and complete post-discharge follow-up are needed to confirm these findings.

## 5 Conclusion

In this single-centre cohort of older adults with Parkinson’s disease hospitalised for lower respiratory tract infection, severe in-hospital deterioration was associated with general comorbidity burden, hypoxaemia, hypoalbuminaemia, sepsis, and pre-admission mobility dependence. Most other available Parkinson’s disease–specific measures did not show clear independent associations, and the study does not establish a Parkinson’s disease–specific prediction strategy. Among survivors, the secondary analysis describes documented return to the index hospital rather than complete population-level readmission. Prospective multicentre studies with standardised phenotyping, appropriate comparison groups, and complete post-discharge follow-up are needed to confirm these findings.

## Data Availability

The raw data supporting the conclusions of this article will be made available by the authors, without undue reservation.
