# Supplementary material for: In-hospital outcomes and documented 90-day readmission to the index hospital in older patients with Parkinson’s disease hospitalised for lower respiratory tract infection: a single-centre retrospective cohort study
Source: Front Neurol. 2026 Sep 16;17:1861214. doi: 10.3389/fneur.2026.1861214 (PMC13623605; doi:10.3389/fneur.2026.1861214)
Supplement: Supplementary file 1 [file Data_Sheet_1.docx]

**Supplementary Material**

*In-hospital outcomes and documented 90-day readmission to the index hospital in older patients with Parkinson’s disease hospitalised for lower respiratory tract infection: a single-centre retrospective cohort study*

**Supplementary Table S1. Distribution of short-term outcomes according to calendar period**

| **Outcome** | **2018–2019 (n = 60)** | **2020–2022 (n = 78)** | **2023–2025 (n = 108)** | **P value** |
| --- | --- | --- | --- | --- |
| Composite adverse in-hospital outcome, n/N (%) | 16/60 (26.7) | 23/78 (29.5) | 23/108 (21.3) | 0.427 |
| ICU admission, n/N (%) | 12/60 (20.0) | 17/78 (21.8) | 17/108 (15.7) | 0.554 |
| Invasive mechanical ventilation, n/N (%) | 7/60 (11.7) | 10/78 (12.8) | 11/108 (10.2) | 0.853 |
| In-hospital death, n/N (%) | 5/60 (8.3) | 6/78 (7.7) | 7/108 (6.5) | 0.896 |
| Discharged alive, n/N (%) | 55/60 (91.7) | 72/78 (92.3) | 101/108 (93.5) | 0.896 |
| Non-home discharge among survivors, n/N (%) | 18/55 (32.7) | 27/72 (37.5) | 40/101 (39.6) | 0.697 |
| Documented 90-day readmission to the index hospital among survivors, n/N (%) | 19/55 (34.5) | 25/72 (34.7) | 30/101 (29.7) | 0.731 |

Values are n/N (%). Comparisons across calendar periods were performed using the χ² test or Fisher’s exact test, as appropriate. Calendar periods were defined according to the date of the index admission. The post-discharge analysis included only patients discharged alive. ICU, intensive care unit.

**Supplementary Table S2. Sensitivity analyses for composite adverse in-hospital outcomes accounting for calendar period**

| **Variable** | **Primary model, adjusted OR (95% CI)** | **P value** | **Calendar-period-adjusted model, adjusted OR (95% CI)** | **P value** | **Excluding 2020–2022 admissions, adjusted OR (95% CI)** | **P value** |
| --- | --- | --- | --- | --- | --- | --- |
| Age, per 1-year increase | 1.04 (0.99–1.10) | 0.089 | 1.04 (0.99–1.10) | 0.145 | 1.05 (0.98–1.12) | 0.152 |
| Charlson Comorbidity Index, per 1-point increase | 1.22 (1.03–1.45) | 0.021 | 1.21 (1.02–1.44) | 0.030 | 1.24 (1.00–1.54) | 0.051 |
| Clinical Frailty Scale ≥5 | 1.58 (0.84–2.96) | 0.153 | 1.54 (0.81–2.92) | 0.187 | 1.62 (0.73–3.59) | 0.235 |
| SpO₂ on room air <92% at admission | 2.05 (1.05–4.01) | 0.036 | 2.01 (1.02–3.97) | 0.044 | 2.12 (0.96–4.68) | 0.063 |
| Serum albumin <35 g/L | 3.45 (1.83–6.52) | <0.001 | 3.39 (1.78–6.47) | <0.001 | 3.52 (1.63–7.59) | 0.001 |
| Aspiration-related LRTI | 1.90 (1.02–3.55) | 0.041 | 1.86 (0.99–3.49) | 0.054 | 1.92 (0.85–4.33) | 0.116 |
| Sepsis or septic shock at admission | 2.58 (1.27–5.23) | 0.009 | 2.51 (1.22–5.16) | 0.012 | 2.64 (1.10–6.34) | 0.030 |
| Calendar period: 2020–2022 vs 2018–2019 | Not included | — | 1.18 (0.53–2.64) | 0.686 | Not applicable | — |
| Calendar period: 2023–2025 vs 2018–2019 | Not included | — | 0.78 (0.35–1.72) | 0.541 | 0.82 (0.35–1.93) | 0.649 |
| Number of patients | 246 | — | 246 | — | 168 | — |
| Number of composite events | 62 | — | 62 | — | 39 | — |
| C-statistic (95% CI) | 0.82 (0.76–0.87) | — | 0.83 (0.77–0.88) | — | 0.83 (0.76–0.90) | — |

The primary model corresponds to the analysis reported in Table 3. The calendar-period-adjusted model additionally included calendar period as a three-level categorical covariate, with 2018–2019 as the reference category. The period-restricted analysis excluded admissions occurring during 2020–2022. Estimates from the period-restricted model should be interpreted cautiously because only 39 composite events were available. OR, odds ratio; CI, confidence interval; LRTI, lower respiratory tract infection; SpO₂, peripheral oxygen saturation.

**Supplementary Table S3. Sensitivity analyses for documented 90-day readmission to the index hospital accounting for calendar period**

| **Variable** | **Primary model, adjusted HR (95% CI)** | **P value** | **Calendar-period-adjusted model, adjusted HR (95% CI)** | **P value** | **Excluding 2020–2022 admissions, adjusted HR (95% CI)** | **P value** |
| --- | --- | --- | --- | --- | --- | --- |
| Age, per 1-year increase | 1.01 (0.98–1.05) | 0.412 | 1.01 (0.98–1.05) | 0.572 | 1.02 (0.98–1.07) | 0.377 |
| Charlson Comorbidity Index, per 1-point increase | 1.13 (1.01–1.27) | 0.033 | 1.12 (1.00–1.26) | 0.055 | 1.15 (0.99–1.34) | 0.070 |
| Serum albumin <35 g/L at admission | 1.82 (1.12–2.95) | 0.016 | 1.79 (1.10–2.92) | 0.019 | 1.88 (1.05–3.37) | 0.034 |
| Prior hospitalisation for pneumonia within 12 months | 1.69 (1.05–2.73) | 0.031 | 1.66 (1.02–2.69) | 0.040 | 1.71 (0.96–3.04) | 0.068 |
| Composite adverse in-hospital outcome | 1.78 (1.07–2.97) | 0.026 | 1.74 (1.04–2.92) | 0.035 | 1.81 (0.98–3.35) | 0.058 |
| Length of stay, per 1-day increase | 1.03 (1.00–1.06) | 0.048 | 1.03 (1.00–1.06) | 0.047 | 1.03 (0.99–1.07) | 0.136 |
| Discharge to non-home setting | 1.96 (1.19–3.24) | 0.008 | 1.91 (1.15–3.18) | 0.013 | 2.02 (1.11–3.67) | 0.021 |
| Aspiration-related LRTI | 1.43 (0.88–2.32) | 0.147 | 1.40 (0.86–2.28) | 0.176 | 1.47 (0.79–2.74) | 0.225 |
| Calendar period: 2020–2022 vs 2018–2019 | Not included | — | 1.05 (0.58–1.89) | 0.871 | Not applicable | — |
| Calendar period: 2023–2025 vs 2018–2019 | Not included | — | 0.86 (0.48–1.54) | 0.612 | 0.88 (0.47–1.65) | 0.690 |
| Number of patients discharged alive | 228 | — | 228 | — | 156 | — |
| Number of documented readmissions | 74 | — | 74 | — | 49 | — |
| Harrell’s C-index (95% CI) | 0.73 (0.67–0.79) | — | 0.73 (0.67–0.79) | — | 0.74 (0.67–0.81) | — |

The primary model corresponds to the analysis reported in Table 4. The calendar-period-adjusted model additionally included calendar period as a three-level categorical covariate, with 2018–2019 as the reference category. The period-restricted analysis excluded admissions occurring during 2020–2022. Estimates from the period-restricted model should be interpreted cautiously because of the reduced sample size and number of readmission events. HR, hazard ratio; CI, confidence interval; LRTI, lower respiratory tract infection.

**Supplementary Table S4. Additional Parkinson’s disease–related sensitivity analyses for composite adverse in-hospital outcomes**

| **PD-related variable added separately to the base model** | **Adjusted OR (95% CI)** | **P value** |
| --- | --- | --- |
| Parkinson’s disease duration, per 1-year increase | 1.05 (0.98–1.11) | 0.155 |
| Tube feeding at admission | 1.57 (0.76–3.23) | 0.222 |
| ≥3 antiparkinsonian medications | 1.17 (0.60–2.28) | 0.641 |
| Prior antipsychotic use for hallucinations or psychosis | 1.34 (0.64–2.82) | 0.439 |

Each Parkinson’s disease–related variable was added separately to the base logistic regression model containing age, Charlson Comorbidity Index, Clinical Frailty Scale ≥5, SpO₂ <92%, serum albumin <35 g/L, aspiration-related LRTI, and sepsis or septic shock. The variables were not entered simultaneously because of clinical overlap, potential collinearity, and the limited number of composite outcome events. OR, odds ratio; CI, confidence interval; PD, Parkinson’s disease; LRTI, lower respiratory tract infection; SpO₂, peripheral oxygen saturation.

**Supplementary Table S5. Exploratory associations of Parkinson’s disease–related characteristics with documented 90-day readmission to the index hospital**

| **PD-related variable added separately to the exploratory Cox model** | **Adjusted HR (95% CI)** | **P value** |
| --- | --- | --- |
| Hoehn–Yahr stage 3–5 | 1.24 (0.74–2.08) | 0.412 |
| Pre-admission wheelchair- or bed-bound status | 1.46 (0.90–2.37) | 0.123 |
| Parkinson’s disease duration, per 1-year increase | 1.02 (0.97–1.07) | 0.487 |
| Tube feeding at admission | 1.31 (0.78–2.21) | 0.304 |
| ≥3 antiparkinsonian medications | 1.10 (0.68–1.78) | 0.696 |

Each Parkinson’s disease–related variable was added separately to the exploratory Cox proportional hazards model reported in Table 4. The model included age, Charlson Comorbidity Index, serum albumin <35 g/L, prior pneumonia-related hospitalisation, composite adverse in-hospital outcome, length of stay, non-home discharge, and aspiration-related LRTI. HR, hazard ratio; CI, confidence interval; PD, Parkinson’s disease; LRTI, lower respiratory tract infection.

**Supplementary Table S6. Sensitivity analysis of invasive mechanical ventilation or in-hospital death as a harder in-hospital endpoint**

| **Variable** | **Adjusted OR** | **95% CI** | **P value** |
| --- | --- | --- | --- |
| Age, per 1-year increase | 1.04 | 0.98–1.10 | 0.193 |
| Charlson Comorbidity Index, per 1-point increase | 1.21 | 1.00–1.46 | 0.047 |
| Clinical Frailty Scale ≥5 | 1.34 | 0.63–2.86 | 0.449 |
| SpO₂ on room air <92% at admission | 2.28 | 1.06–4.92 | 0.035 |
| Serum albumin <35 g/L | 3.54 | 1.65–7.57 | 0.001 |
| Aspiration-related LRTI | 1.68 | 0.78–3.63 | 0.185 |
| Sepsis or septic shock at admission | 3.02 | 1.33–6.86 | 0.008 |
| Hoehn–Yahr stage 3–5 | 1.46 | 0.63–3.40 | 0.376 |
| Pre-admission wheelchair- or bed-bound status | 1.92 | 0.89–4.13 | 0.096 |
| Number of patients | 246 | — | — |
| Number of hard-endpoint events | 38 | — | — |
| C-statistic | 0.85 | 0.79–0.91 | — |

The sensitivity endpoint was defined as invasive mechanical ventilation or in-hospital death during the index admission. Components were not mutually exclusive. Estimates were obtained using Firth penalised logistic regression with the same covariates as the Parkinson’s disease–specific extended model. This analysis was performed to reduce dependence on institution-specific ICU admission decisions. OR, odds ratio; CI, confidence interval; LRTI, lower respiratory tract infection; SpO₂, peripheral oxygen saturation.
